# Genomic Analysis Revealed New Oncogenic Signatures in *TP53*-Mutant Hepatocellular Carcinoma

**DOI:** 10.3389/fgene.2018.00002

**Published:** 2018-02-02

**Authors:** Venkatesh Kancherla, Samir Abdullazade, Matthias S. Matter, Manuela Lanzafame, Luca Quagliata, Guglielmo Roma, Yujin Hoshida, Luigi M. Terracciano, Charlotte K. Y. Ng, Salvatore Piscuoglio

**Affiliations:** ^1^Institute of Pathology, University Hospital Basel, Basel, Switzerland; ^2^Department of Biology, University of Naples Federico II, Naples, Italy; ^3^Division of Liver Diseases, Department of Medicine, Liver Cancer Program, Tisch Cancer Institute, Graduate School of Biomedical Sciences, Icahn School of Medicine at Mount Sinai, New York, NY, United States; ^4^Department of Biomedicine, University of Basel, Basel, Switzerland

**Keywords:** *TP53* mutations, somatic mutations, copy number alterations, mutational signature, oncogenic signature

## Abstract

The *TP53* gene is the most commonly mutated gene in human cancers and mutations in *TP53* have been shown to have either gain-of-function or loss-of-function effects. Using the data generated by The Cancer Genome Atlas, we sought to define the spectrum of *TP53* mutations in hepatocellular carcinomas (HCCs) and their association with clinicopathologic features, and to determine the oncogenic and mutational signatures in *TP53*-mutant HCCs. Compared to other cancer types, HCCs harbored distinctive mutation hotspots at V157 and R249, whereas common mutation hotspots in other cancer types, R175 and R273, were extremely rare in HCCs. In terms of clinicopathologic features, in addition to the associations with chronic viral infection and high Edmondson grade, we found that *TP53* somatic mutations were less frequent in HCCs with cholestasis or tumor infiltrating lymphocytes, but were more frequent in HCCs displaying necrotic areas. An analysis of the oncogenic signatures based on the genetic alterations found in genes recurrently altered in HCCs identified four distinct *TP53*-mutant subsets, three of which were defined by *CTNNB1* mutations, 1q amplifications or 8q24 amplifications, respectively, that co-occurred with *TP53* mutations. We also found that mutational signature 12, a liver cancer-specific signature characterized by T>C substitutions, was prevalent in HCCs with wild-type *TP53* or with missense *TP53* mutations, but not in HCCs with deleterious *TP53* mutations. Finally, whereas patients with HCCs harboring deleterious *TP53* mutations had worse overall and disease-free survival than patients with *TP53*-wild-type HCCs, patients with HCCs harboring missense *TP53* mutations did not have worse prognosis. In conclusion, our results highlight the importance to consider the genetic heterogeneity among *TP53*-mutant HCCs in studies of biomarkers and molecular characterization of HCCs.

## Introduction

Hepatocellular carcinomas (HCCs) display extensive histologic, transcriptomic and genetic diversity (Lee et al., 2004; Boyault et al., 2007; Chiang et al., 2008; Hoshida et al., 2009; Fujimoto et al., 2012; Guichard et al., 2012; Ahn et al., 2014; Schulze et al., 2015; The Cancer Genome Atlas Research Network, 2017). On the genetic level, genes involved in liver metabolism, Wnt and p53 signaling have been shown to be recurrently altered (Fujimoto et al., 2012; Guichard et al., 2012; Ahn et al., 2014; Schulze et al., 2015; The Cancer Genome Atlas Research Network, 2017). The most frequently mutated protein-coding genes are *CTNNB1* (encoding β-catenin) and *TP53* (encoding p53), both mutated in 20–40% of HCCs (Fujimoto et al., 2012; Guichard et al., 2012; Ahn et al., 2014; Schulze et al., 2015; The Cancer Genome Atlas Research Network, 2017).

*TP53* is the most frequently mutated gene in human cancers (Kandoth et al., 2013). The p53 protein modulates multiple cellular functions, including transcription, DNA synthesis and repair, cell cycle arrest, senescence and apoptosis (Vogelstein et al., 2000). Mutations in *TP53* can abrogate these functions, leading to genetic instability and progression to cancer (Vogelstein et al., 2000). Across 12 major cancer types (excluding HCC), 42% of cancers harbored *TP53* somatic mutations, with at least 20% mutational rate in 10/12 cancer types and *TP53* mutations are associated with inferior prognosis and unfavorable clinicopathologic parameters, such as tumor stage (Kandoth et al., 2013). Furthermore, *TP53*-mutant tumors are highly enriched among tumors driven by copy number alterations (CNAs), with most remaining *TP53*-mutant tumors associated with the presence of somatic mutations in the Wnt and/or the RAS-RAF-ERK signaling pathways (Ciriello et al., 2013).

The pattern of *TP53* mutations is reminiscent of both an oncogene and a tumor suppressor gene (Vogelstein et al., 2013). The majority (86%) of *TP53* mutations are in the DNA-binding domain (Olivier et al., 2010; Kandoth et al., 2013). Most mutations in the DNA-binding domain are missense (88%) and approximately 1/3 of missense mutations affect the hotspot residues R175, G245, R248, R249, R273, and R282 (Olivier et al., 2010). Outside the DNA-binding domain, most mutations (∼60%) are nonsense or frameshift (Olivier et al., 2010). Mutant p53 proteins may lose the tumor suppressive functions and exert dominant-negative activities, but may also gain new oncogenic properties (Olivier et al., 2010; Muller and Vousden, 2014). Indeed, on the immunohistochemical level, p53 is generally detectable to various extents in samples with missense mutations but is undetectable in samples with truncating or frameshift mutations (Hall and McCluggage, 2006; Soussi et al., 2014).

In HCC, *TP53* mutational frequency has been reported to range between 22 and 33% (Fujimoto et al., 2012; Guichard et al., 2012; Cleary et al., 2013; Kan et al., 2013; Ahn et al., 2014; Jhunjhunwala et al., 2014; Shiraishi et al., 2014; Totoki et al., 2014; Weinhold et al., 2014; Schulze et al., 2015; Fujimoto et al., 2016; The Cancer Genome Atlas Research Network, 2017). However, the frequency varies between geographic regions, etiological factors and carcinogen exposure, with more frequent *TP53* mutations in regions where hepatitis B virus (HBV) infection is endemic (Fujimoto et al., 2012; Guichard et al., 2012; The Cancer Genome Atlas Research Network, 2017). Similar to other cancer types, *TP53*-mutant HCCs have been associated with features linked to poor prognosis, including high levels of alpha-fetoprotein, high Edmondson grade, expression of stem-like markers, and activation of pro-oncogenic signaling pathways (Kiani et al., 2002; Breuhahn et al., 2004; Lee et al., 2004; Peng et al., 2004; Boyault et al., 2007; Chiang et al., 2008; Hoshida et al., 2009; Goossens et al., 2015). Furthermore, patients with *TP53*-mutant HCCs tend to have shorter overall (OS) and disease-free survival (DFS) (Yano et al., 2007; Woo et al., 2011; Cleary et al., 2013). However, it appears that not all *TP53* mutations in HCCs are equal. For instance, one of the most common mutation hotspots affecting residues R248/249 has an overall frequency of ∼10% among *TP53*-mutant HCCs (Fujimoto et al., 2012, 2016; Ahn et al., 2014; Schulze et al., 2015; The Cancer Genome Atlas Research Network, 2017). In particular, the R249S mutation resulting from G>T transversion has specifically been linked to the combined effect of aflatoxin B1 exposure and HBV infection (Bressac et al., 1991; Hsu et al., 1991) and this mutation is detected in >75% of HCC from areas with high aflatoxin B1 exposure (Gouas et al., 2009; Kew, 2010). Further hotspot mutations affecting preferentially HCC are located at the residues V157 and H193 (both at ∼2%) (Fujimoto et al., 2012, 2016; Ahn et al., 2014; Schulze et al., 2015; The Cancer Genome Atlas Research Network, 2017). Both R249S and V157F have been associated with stem cell-like traits and poor prognosis in HCC patients (Villanueva and Hoshida, 2011; Woo et al., 2011).

Finally, molecular classification studies have invariably grouped *TP53*-mutant HCCs under the umbrella of the aggressive subclass, but it is also clear that this subclass is molecularly, biologically and clinically heterogeneous (Boyault et al., 2007; Hoshida et al., 2009; Goossens et al., 2015).

Given the diverse pattern of *TP53* mutations, taking advantage of The Cancer Genome Atlas (TCGA) dataset, in this study we sought to determine the pattern of *TP53* somatic mutations in HCCs and its association with clinicopathologic features. Additionally, as *TP53* mutations are associated with HCC molecular subclasses with poor prognosis, we sought to define the oncogenic and mutational signatures among *TP53*-mutant HCCs.

## Materials and Methods

### Sample Selection and Histologic Assessment

From TCGA liver hepatocellular carcinoma (LIHC) project (The Cancer Genome Atlas Research Network, 2017), 373 tumors with available somatic mutational data^1^ (accessed April 2017) (Gao et al., 2013) were included in the study. Images of diagnostic hematoxylin & eosin (H&E) slides were retrieved from the cbioportal and reviewed by three expert hepatopathologists (SA, MSM and LMT) according to the guidelines by the World Health Organization (Bosman et al., 2010) to define the presence or absence of cholestasis, Mallory bodies, tumor infiltrating lymphocytes (TILs), vessel infiltration and necrotic areas. 4-point scale Edmondson and Steiner system was adopted for tumor grading as previously described (Edmondson and Steiner, 1954; Alexandrov et al., 2013). Clinical information were obtained from the cbioportal (Gao et al., 2013).

### Classification of *TP53* Somatic Mutations

*TP53* somatic non-synonymous and splice region mutations for the 373 HCCs were retrieved from the cbioportal (accessed April 2017) (Gao et al., 2013). *TP53* mutations were stratified according to (i) the mutation type as single-nucleotide missense mutations (also encompassing synonymous mutations affecting splice region, Supplementary Methods and Supplementary Table S1) or deleterious mutations (encompassing splice site, nonsense, in-frame, and frameshift mutations); (ii) whether the mutations were within or outside of the DNA-binding domain. For correlative analyses with clinicopathologic parameters, the sample (TCGA-DD-A1EE) with three *TP53* mutations (A161S, H193R and C277^∗^) was classified as harboring deleterious mutation.

The spectrum of *TP53* mutations in non-LIHC TCGA datasets were retrieved from the cbioportal (accessed June 2017, Supplementary Table S2) (Gao et al., 2013). Mutation (lolliplot) diagrams and Oncoprints were generated using cbioportal (Gao et al., 2013).

### Genomic and Transcriptomic Data Analysis

Gene-level copy number (“gistic2_thresholded,” 370/373 samples) and expression (“IlluminaHiSeq,” 367/373 samples) data were retrieved from the UCSC Xena Functional Genomics Browser^2^ accessed April 2017). Gene-level copy number data were used to define genomic regions with differential frequencies of copy number alterations between HCCs with missense *TP53* mutations, with deleterious *TP53* mutations, or with wild-type *TP53*. Copy number states -2, -1, 0, 1, and 2 were considered homozygous deletion, heterozygous loss, copy number neutral, gain and high-level gain/amplification, respectively.

Transcriptomic data were in the form of gene-level, log-transformed, upper-quartile-normalized RSEM values. Molecular classification was performed according to Hoshida et al. (2009), using the Nearest Template Prediction: http://software.broadinstitute.org/cancer/software/genepattern. The R package limma was used to perform quantile normalization and for differential expression analysis. Multiple correction was performed using the Benjamini–Hochberg method. Genes with adjusted *P*-value < 0.05 were considered as differentially expressed.

The number of somatic mutations per sample was obtained from the cbioportal (Gao et al., 2013).

### Oncogenic Signatures

Oncogenic signature (“oncosign”) classification and the selection of genomic features as ‘selected functional elements’ (SFEs) input data were performed as described by Ciriello et al. (2013). Specifically, we selected 29 significantly mutated genes that have previously been reported as cancer genes (Futreal et al., 2004; Fujimoto et al., 2012; Kandoth et al., 2013; Lawrence et al., 2014), 27 recurrent amplifications and 34 recurrent deletions as SFEs (Supplementary Methods). Robustness of the subclasses was assessed by removing 5, 10, or 20% of samples, reclassifying the reduced dataset, and calculating the Jaccard coefficients over 20 runs (Ciriello et al., 2013). Enrichment of genomic alterations was assessed using Chi-squared and Fisher’s exact tests, as described (Ciriello et al., 2013).

### Mutational Signatures

Decomposition of mutational signatures was performed using deconstructSigs (Rosenthal et al., 2016), based on the set of 30 mutational signatures (“signature.cosmic”) (Alexandrov et al., 2013; Nik-Zainal et al., 2016), for the 358 samples with at least 30 somatic mutations. Mutational signatures with >20% weight were considered to have substantial contribution to the overall mutational landscape. For each sample, the mutation signature with the highest weight was considered the dominant mutational signature.

### Pathway Analysis

Pathways analysis was performed using Ingenuity Pathway Analysis as previously described (Piscuoglio et al., 2014; Martelotto et al., 2015). *P <* 0.001 was considered significant (Supplementary Methods).

### Statistical Analysis

Associations between *TP53* mutations and clinical/histologic features were assessed using Mann–Whitney *U*, Chi-squared or Fisher’s exact tests as appropriate. Survival analyses were performed using the Kaplan-Meier method and the log-rank test. Univariate and multivariate analyses for OS and DFS were performed using the Cox proportional-hazards model. Mutual exclusivity and co-occurrence of somatic mutations were defined using the cbioportal (Gao et al., 2013). Statistical analyses comparing copy number profiles and defining genes up-regulated when gained or amplified and genes down-regulated when lost were performed as previously described (Supplementary Methods) (Piscuoglio et al., 2014). All tests were two-sided. *P <* 0.05 were considered statistically significant. Statistical analyses were performed with R v3.1.2 or SPSS v24 (IBM, Münchenstein, CH).

## Results

### Clinicopathologic Characterization and Molecular Classification of HCCs

*TP53* mutation status was available for 373 HCCs subjected to whole-exome sequencing by TCGA (The Cancer Genome Atlas Research Network, 2017). Analysis of the clinical details of the patients revealed that the median age at diagnosis was 61 (range 16–90) and that 67.5% were male (Supplementary Table S3). Half of the patients were Caucasian (50.8%), with most remaining patients being Asian (43.9%). The most frequent primary risk factor was alcohol consumption (33.1%), followed by HBV (30.0%) and hepatitis C virus (HCV) infection (15.9%). Overall, history of at least one primary risk factor was noted in 74.2% patients (Supplementary Table S3).

**FIGURE 1:**
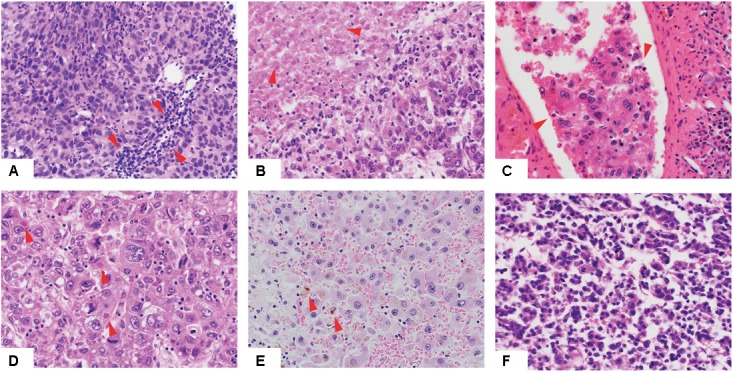
Histologic features of hepatocellular carcinoma. Low-power view of hepatocellular carcinomas with tumor infiltrating lymphocytes **(A)**, necrotic areas **(B)**, vessel infiltration **(C)**, Mallory bodies **(D)**, cholestasis **(E)** and of high Edmondson grade **(F)**. Red arrows indicate the relevant histologic features.

We performed a comprehensive histopathologic review of the diagnostic H&E slides for all 373 included cases to assess Edmondson grade, the presence of cholestasis, Mallory bodies, vessel infiltration, necrotic areas, and TILs (**Figure 1** and Supplementary Table S3). Most samples were of intermediate grade, with 33.2, 60.6, and 5.4% graded as of Edmondson grades 2, 3, and 4, respectively. No sample was classified as of Edmondson grade 1. Cholestasis, Mallory bodies, vessel infiltration, necrotic areas, and TILs were present in 21.6, 22.0, 34.1, 24.8, and 47.3% of cases, respectively.

Molecular classification was performed for the 367 HCCs for which expression data were available according to Hoshida et al. (2009). 31.3, 21.5, and 47.2% of HCCs were classified as S1, S2 and S3, respectively (Supplementary Table S3).

### Spectrum of *TP53* Somatic Mutations in HCCs

**FIGURE 2:**
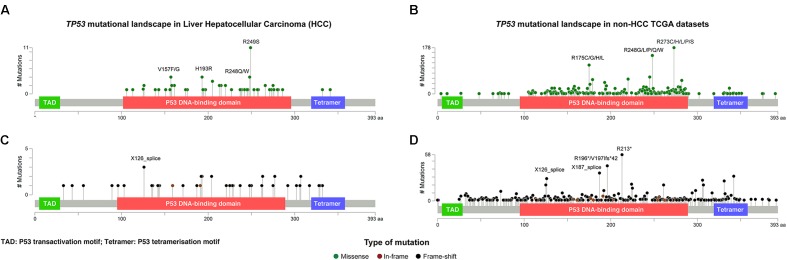
The distribution and the spectrum of *TP53* mutations. The distribution and spectrum of *TP53* missense **(A,B)** and deleterious **(C,D)** mutations in hepatocellular carcinoma **(A,C)** and in non-liver TCGA datasets **(B,D)**. Diagrams represent the protein domains of p53 encoded by the *TP53* gene. The presence of a mutation is shown on the *x* axis (lollipop), and the frequency of mutations is shown on the *y* axis. Missense mutations are presented as green circles, deleterious mutations (i.e., nonsense, frameshift, splice-site and in-frame) are depicted in black and brown circles. Plots were generated using cBioPortal tools (http://www.cBioPortal.org) and curated manually.

Given that *TP53* is one of the most frequently mutated genes in HCCs and its diverse spectrum of mutations in human cancers, we sought to define the spectrum and type of *TP53* mutations found in HCCs. A total of 116 somatic non-synonymous *TP53* mutations and 2 synonymous *TP53* mutations affecting splice regions were identified in 115 (30.8%) cases, including one case with three distinct mutations and one case with two. Missense (including missense and synonymous mutations affecting splice region, Supplementary Methods and Supplementary Table S1) and deleterious (including nonsense, frame-shift, in-frame, splice site) mutations accounted for 73 (62%) and 45 (38%), respectively (**Figure 2**). Compared to other cancer types characterized by the TCGA, there was no difference between HCC and non-HCC tumor types in terms of the ratio of missense vs deleterious mutations (*P* = 0.197, Fisher’s exact test).

Of the 73 missense and synonymous mutations affecting splice region, 51 (70%) affected known hotspot residues (Chang et al., 2016; Gao et al., 2017) and all but one (99%) affected the DNA-binding domain (**Figure 2A**). All missense mutations were predicted to be pathogenic by at least 2/5 *in silico* mutation effect predictors, with the two synonymous mutations affecting splice region also predicted to be disease causing (Supplementary Methods and Supplementary Table S1). The most frequent hotspot mutations were R249S (11/73, 15%), H193R (4/73, 5%), and R248Q/W (4/73, 5%). V157F, a mutation not considered to be a hotspot residue (Chang et al., 2016; Gao et al., 2017) but was reported as a mutation hotspot in HCCs (Woo et al., 2011), accounted for 4/73 (5%) of the missense mutations (**Figure 2A**). Compared to other cancer types, mutations affecting V157 and R249 accounted for greater proportions of the missense mutations in HCCs than in other cancer types (4/73, 5% vs. 22/1787, 1.2%, *P* = 0.017 and 11/73, 15% vs. 21/1787, 1.2%, *P <* 0.001, respectively, Fisher’s exact tests, **Figures 2A,B**). In particular, R249S accounted for <0.5% of *TP53* missense mutations in non-HCC TCGA samples, but accounted for 15% of the missense mutations in HCCs (*P <* 0.001, Fisher’s exact test). In contrast, the most frequent hotspots in non-HCC tumors R273 (178/1787, 10.0% of missense mutations) and R175 (112/1787, 6.3%) were only observed once and not at all, respectively, in HCCs (*P* = 0.008 and *P* = 0.020, respectively, Fisher’s exact tests).

The 45 deleterious mutations comprised 13 (29%) nonsense point mutations, 20 (44%) frameshift small insertions or deletions (indels), 3 (7%) in-frame indels and 9 (20%) mutations affecting splice sites. Unlike missense mutations, the 45 deleterious mutations were spread across the *TP53* gene, with 32 (71%) in the DNA-binding domain, 3 (7%) in the tetramerization motif and 10 (22%) outside of these two domains (**Figure 2C**). In other cancer types, recurrent truncating mutations were observed at R196 (44/926, 4.8% of deleterious *TP53* mutations) and R213 (56/926, 6.0%), both of which were not observed in HCC (**Figures 2C,D**).

Our results demonstrate that the spectrum of *TP53* mutations in HCCs is distinct from that in non-HCC tumors, with HCC-specific recurrent hotspot mutations and a near absence of highly recurrent *TP53* mutations found in other cancer types.

### *TP53* Status Correlates with Specific Histopathologic and Clinical Features of HCCs

Next, we sought to define whether *TP53* mutation status correlated with clinicopathologic parameters. *TP53* mutations were more frequently found in male patients (35.9% vs. 20.7% in female; *P* = 0.003, Fisher’s exact test) and in patients with at least one primary risk factor (35.1% vs. 20.9%; *P* = 0.013, Fisher’s exact test), especially in HCCs associated with HBV/HCV infection (53.1% vs. 39.7%; *P* = 0.021, Fisher’s exact test, **Table 1**). Patients from different racial backgrounds were associated with different *TP53* mutational frequencies (*P* = 0.001, Chi-squared test, **Table 1**). Black or African Americans had the highest frequency of *TP53* mutations (70.6% vs. Asians, 36.5%, *P* = 0.009, and vs. Caucasians, 22.8%, *P <* 0.001, Fisher’s exact tests), while Asians displayed more frequent *TP53* mutations than Caucasians (*P* = 0.006, Fisher’s exact test). No association with age of patients or Child-Pugh classification was observed.

**Table 1:** Analyses of *TP53* status and clinicopathologic parameters in the 373 HCCs from The Cancer Genome Atlas cohort (The Cancer Genome Atlas Research Network, 2017).

		*TP53* status		*P*-value
		Mutant [*N* (%)]	Wild-type [*N* (%)]
Age (*n* = 372)	Median years	59	61	0.200
Gender (*n* = 372)	Female	25 (20.7)	96 (79.3)	**0.003**
	Male	90 (35.9)	161 (64.1)
Child-Pugh classification grade (*n* = 243)	A	65 (29.4)	156 (70.6)	0.754
	B	7 (33.3)	14 (66.7)
	C	0 (0)	1 (100)
Race (*n* = 362)	America Indian or Alaskan native	1 (50)	1 (50)	**<0.001**
	Asian	58 (36.5)	101 (63.5)
	Black or African American	12 (70.6)	5 (29.4)
	Caucasian	42 (22.8)	142 (77.2)
History of Primary Risk Factors (*n* = 353)	At least one risk factor	92 (35.1)	170 (64.9)	**0.013**
	No risk factor	19 (20.9)	72 (79.1)
Edmondson Grade (*n* = 373)	2	15 (12.1)	109 (87.9)	**<0.001**
	3	87 (38.5)	139 (61.5)
	4	13 (65.0)	7 (35.0)
Cholestasis (*n* = 370)	Absent	101 (38.4)	189 (65.2)	**0.003**
	Present	14 (17.5)	66 (82.5)
Mallory Bodies (*n* = 373)	Absent	94 (32.3)	197 (67.7)	0.280
	Present	21 (25.6)	61 (74.4)
Vessel infiltration (*n* = 370)	Absent	72 (29.5)	172 (70.5)	0.407
	Present	43 (34.1)	83 (65.9)
Necrotic areas (*n* = 371)	Absent	75 (26.9)	204 (73.1)	**0.004**
	Present	40 (43.5)	52 (56.5)
Infiltrating lymphocytes (*n* = 372)	Absent	72 (62.6)	124 (48.2)	**0.013**
	Present	43 (37.4)	133 (51.8)
Molecular classification by Hoshida et al. (2009, *n* = 367)	S1	42 (36.5)	73 (63.5)	**0.001**
	S2	31 (42.5)	42 (57.5)
	S3	39 (21.8)	140 (78.2)

*Statistical comparisons were performed using Mann–Whitney *U* test, Fisher’s exact test or Chi-Squared test. *P* < 0.05 was considered to be statistically significant*.

Correlation with histologic features revealed that *TP53*-mutant HCCs were associated with high Edmondson grade, accounting for 12.1, 38.5, and 65.0% of cases classified as Edmondson grades 2, 3, and 4, respectively (*P <* 0.001, Chi-squared test, **Table 1**). *TP53* mutations were less frequent in HCCs associated with cholestasis (17.5% vs. 38.4%; *P* = 0.003, Fisher’s exact test) and were more frequent in HCCs with necrotic areas (43.5% vs. 26.9%; *P* = 0.004, Fisher’s exact test, **Table 1**). The presence of TILs was associated with less frequent *TP53* mutations (37.4% vs. 62.6%; *P* = 0.013, Fisher’s exact test; **Table 1**). No association was found between *TP53* mutation status and the presence of Mallory Bodies or vessel infiltration.

Further analyses comparing HCCs with missense or deleterious mutations showed that patients with HCCs with deleterious *TP53* mutations were slightly older than those with missense mutations (median 64 vs. 58, *P* = 0.049, Mann–Whitney *U* test, Supplementary Table S4). After excluding one patient (TCGA-DD-A1EE) with both deleterious mutation (C277^∗^) and hotspot missense (H193R) mutations, the ages between the two groups were not different (*P* = 0.058, Mann–Whitney *U* test). Of note, *TP53* recurrent hotspots V157F, R158H, H193R, Y205, and R249S were exclusively found in tumors of high Edmondson grade (grades 3/4, *P* = 0.038, Fisher’s exact test, compared to HCCs with other *TP53* mutations).

Correlating *TP53* status with molecular classification, (Hoshida et al., 2009) *TP53*-mutant HCCs were preferentially enriched in the S1 and S2 subclasses (36.5% and 42.5% vs. 21.8% in S3, *P* = 0.001, Chi-squared test, **Table 1**). Stratifying *TP53*-mutant HCCs into those with missense or deleterious mutations did not reveal association between *TP53* mutation types and molecular classification (*P* = 0.459, Chi-squared test, Supplementary Table S4).

These results demonstrate that, additional to the well-established associations with the male gender, HBV/HCV infection and high Edmondson grade, *TP53* mutations were less frequent in HCCs with cholestasis or TILs, but were more frequent in HCCs with necrotic areas.

### Genomic Instability Is Not Associated with *TP53* Mutation Type

Next, we compared the number of somatic genetic alterations between *TP53*-wild-type and mutant cases. Mutational burden was higher in *TP53*-mutant HCCs, HCCs with missense *TP53* mutations and HCCs with deleterious *TP53* mutations than *TP53*-wild-type cases (*P <* 0.001, *P <* 0.001 and *P* = 0.004, respectively, Mann–Whitney *U* tests), but no difference was observed between cases with missense or deleterious mutations (*P* = 0.799, Mann–Whitney *U* test, Supplementary Figure S1A). Similarly, *TP53*-mutant HCCs, HCCs with missense *TP53* mutations and HCCs with deleterious *TP53* mutations all harbored higher number of genes affected by CNAs compared with *TP53*-wild-type cases (*P <* 0.001, *P <* 0.001 and *P* = 0.001, respectively, Mann–Whitney *U* tests, Supplementary Figure S1B), with no difference between cases with missense or deleterious *TP53* mutations (*P* = 0.352, Mann–Whitney *U* test, Supplementary Figure S1B).

Consistent with their increased chromosomal instability, *TP53*-mutant HCCs displayed more frequent gains of chromosomes 1p, 3, 10p and 19p and losses of half the genome, notably of chromosomes 4, 5, 10q, 14, 17p, 18 and 19 (Supplementary Figures S2A–C). The CNA landscapes between HCCs with *TP53* missense or deleterious mutations were remarkably similar (Supplementary Figure S2D).

To identify potential CNA drivers associated with *TP53* mutations, we interrogated the genes overexpressed when gained and genes downregulated when lost in the regions that showed differential CNA frequencies between *TP53*-mutant and *TP53*-wild-type cases (Supplementary Figure S2A). Pathway analysis of the copy number-regulated genes revealed that *TP53*-mutant cases displayed deregulation in pathways associated with EIF2 signaling, protein ubiquitination pathway, RNA polymerase-II complex and DNA repair pathways, and in molecular and cellular functions related to cell death and survival, cell cycle, DNA replication, recombination and repair (Supplementary Figure S3).

### *TP53*-Mutant HCCs Displayed Heterogeneous Oncogenic Signatures

**FIGURE 3:**
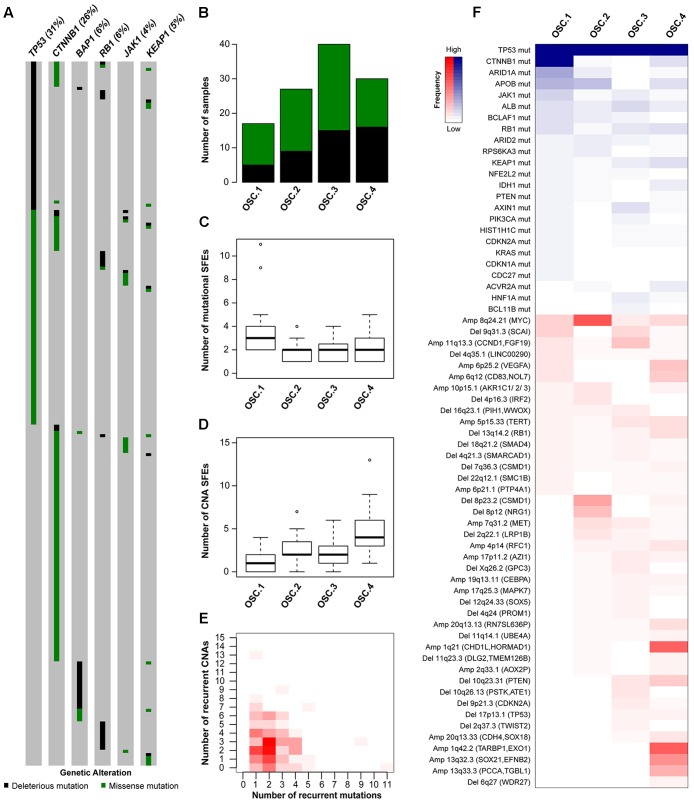
Oncogenic signature classes in *TP53*-mutant hepatocellular carcinoma. The pattern of mutations in *TP53*, *CTNNB1*, *BAP1*, *RB1*, *JAK1* and *KEAP1* in hepatocellular carcinoma **(A)**. Number of *TP53*-mutant samples classified as OSC1, OSC2, OSC3, and OSC4, according to the color key in A **(B)**. Number of mutational **(C)** and copy number **(D)** ‘selected functional elements’ (SFEs) in the different subclasses. The distribution of mutational vs copy number SFEs in *TP53*-mutant cases **(E)**. The shade of red is proportional to the number of samples for a given (*x*,*y*) position. Heatmap shows the mutational and copy number SFEs altered in at least 5% of the samples in at least one oncogenic signature class **(F)**. Shades of red and blue are proportional to the number of samples with a given genetic alteration, according to the color key. Plot in **(A)** was generated using cBioPortal (http://www.cBioPortal.org) and curated manually.

In HCCs, *TP53* and *CTNNB1* mutations were largely mutually exclusive (*P* = 0.028, **Figure 3A**) (Fujimoto et al., 2012; Guichard et al., 2012; Schulze et al., 2015; The Cancer Genome Atlas Research Network, 2017). Additionally, *TP53* and *BAP1* mutations were also mutually exclusive (*P* = 0.004; **Figure 3A**). In contrast, *TP53* mutations co-occurred with *RB1*, *JAK1* and *KEAP1* mutations (*P* = 0.028, *P* = 0.034 and *P* = 0.044, respectively, **Figure 3A**). These observations suggest that *TP53*-mutant HCCs likely constitute a genetically heterogeneous subclass and may be subclassified into categories with distinct oncogenic signatures.

To define the oncogenic signatures in *TP53*-mutant HCCs, we performed unsupervised partitioning of the samples into classes with distinct patterns of likely ‘driver’ genetic alterations (or ‘selected functional elements,’ SFEs), (Ciriello et al., 2013) including mutations in 29 significantly mutated genes, amplifications in 27 recurrently amplified regions, and homozygous deletions in 34 recurrently deleted regions (see Materials and Methods). Among the 144 *TP53*-mutant HCCs with mutational and CNA data, we found median of 2 mutational (range 0–11) and 2.5 CNA (range 0–13) SFEs in each case and identified four robust oncogenic signature classes (OSCs, **Figures 3B–E** and Supplementary Figure S4A). HCCs with missense or deleterious *TP53* mutations did not cluster separately (*P* = 0.305, Chi-squared test, **Figure 3B**), nor HCCs of distinct transcriptomic subclasses (Supplementary Figure S4B).

Inspection of the SFEs that characterized each OSC revealed that OSC1 was defined by the presence of *CTNNB1* mutations (100%, *P* < 0.001, Fisher’s exact test, **Figure 3F**). The most frequent alteration in OSC2 was 8q24.21 amplification (encompassing *MYC*, 67%, *P* < 0.001, Fisher’s exact test), while the most frequent alterations in OSC4 were 1q21.3 (encompassing *CHD1L* and *HORMAD1*, 60%) and 1q42.2 (encompassing *TARBP1* and *EXO1*, 63%) amplifications (both *P* < 0.001, Fisher’s exact tests, **Figure 3F**). OSC3 was notable for lacking highly recurrent genetic alterations, with the most frequent alteration being 11q13.3 amplification (*CCND1*, 23%, *P* = 0.011, Fisher’s exact test). Additionally, *ARID1A* mutations were enriched in OSC1 (35%, *P* < 0.001, Fisher’s exact test), while 10q23.21 deletion (*PTEN*, 20%) and 6p25.2 amplification (*VEGFA*, 23%) were enriched in OSC4 (*P* = 0.020 and *P* = 0.001, respectively, Fisher’s exact tests). We also found that OSC1 harbored higher number of mutational SFEs and lower number of CNA SFEs (*P* < 0.001 and *P* = 0.002, respectively, Mann–Whitney *U* tests, **Figures 3C,D**) compared to other classes. By contrast, OSC4 harbored higher number of CNA SFEs than the other classes (*P* < 0.001, respectively, Mann–Whitney *U* test, **Figure 3D**). The *TP53* R249S hotspot mutation was not associated with specific OSC classes (*P* = 0.591, Chi-squared test). Finally, OSC1/2 were more frequently associated with the presence of TILs than OSC3/4 (*P* = 0.028, Chi-squared test). No other associations between histologic or clinicopathologic parameters and OSCs were found.

These observations are concordant with the observation that tumors are primarily driven by either somatic mutations or CNAs but rarely both (Ciriello et al., 2013) (**Figure 3E** and Supplementary Figures S4C,D). Furthermore, we identified subclasses of *TP53*-mutant HCCs likely driven by co-occurring *CTNNB1* mutations, 8q24.21 (*MYC*) amplification or 1q amplification in a mutually exclusive manner.

### Mutational Signatures in *TP53*-Mutant HCCs

**FIGURE 4:**
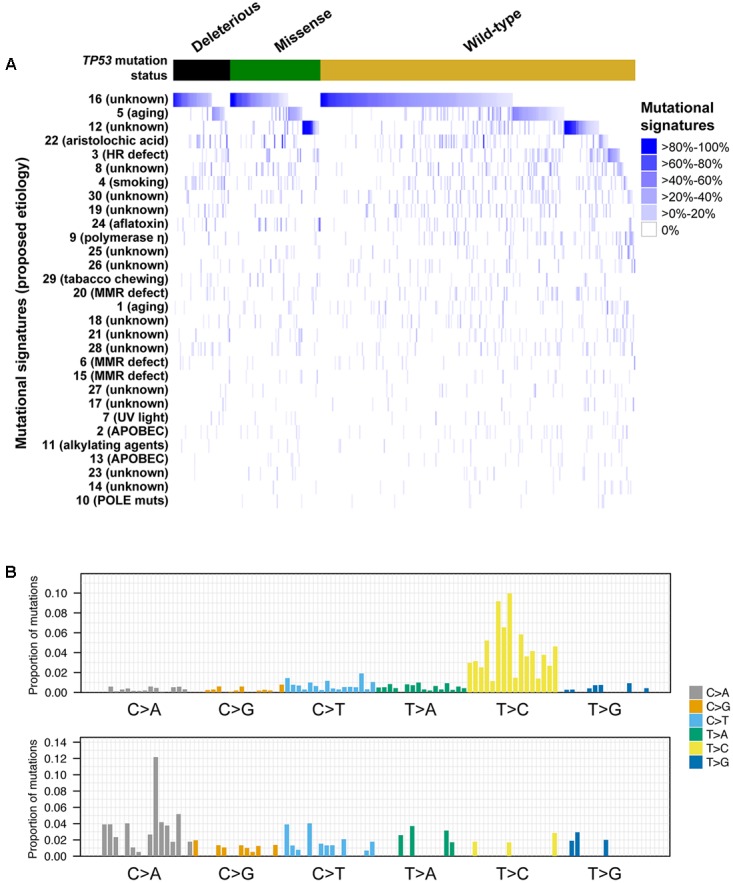
Mutational signatures in hepatocellular carcinoma with and without *TP53* somatic mutations. Heatmap depicting the mutational signatures that shaped the genomes of the tumor samples analyzed **(A)** (Alexandrov et al., 2013). The similarity of the pattern of substitutions to the published mutational signatures is indicated in blue according to the color key. HCC samples were divided according to their *TP53* mutational status. Mutational signatures were sorted by the number of cases classified as having a given mutational signature as the dominant signature, in decreasing order. Barplots illustrating examples of mutational signatures 12 (upper) and 24 (bottom) **(B)**. In each panel, the colored barplot illustrates each mutational signature according to the 96 substitution classification defined by the substitution classes (C>A, C>G, C>T, T>A, T>C, and T>G bins) and the 5′ and 3′ sequence context, normalized using the observed trinucleotide frequency in the human exome to that in the human genome. The bars are ordered first by mutation classes (C>A/G>T, C>G/G>C, C>T/G>A, T>A/A>T, T>C/A>G, T>G/A>C), then by the 5′ flanking base (A, C, G, T) and then by the 3′ flanking base (A, C, G, T).

The somatic mutational landscapes are shaped by endogenous and/or environmental biological and chemical processes (Alexandrov et al., 2013). More than 10 mutational signatures have been identified in liver cancers, including two liver cancer-specific signatures 12 and 16 of unknown etiology, both of which are characterized by frequent T>C substitutions but with different sequence contexts (Alexandrov et al., 2013). To determine whether *TP53*-mutant HCCs harbored distinct mutational signatures compared to *TP53*-wild-type HCCs, we inferred the underlying mutational processes for the 358 HCCs with at least 30 somatic mutations (Alexandrov et al., 2013; Nik-Zainal et al., 2016). The age-associated signature 5, (Alexandrov et al., 2015) and the liver cancer-specific signatures 12 and 16 contributed substantially (≥20% weight) to the mutational landscapes in 17.0, 12.8, and 53.4% of the samples, respectively (**Figure 4**). Together, 72.9% of HCCs harbored signatures 5, 12 or 16 as the dominant signatures (14.0, 10.6, and 48.3%, respectively).

A comparison of the mutational signatures with substantial contribution (≥20%) to the mutational landscapes of *TP53*-mutant or *TP53*-wild-type HCCs revealed that only the aflatoxin-associated signature 24 was enriched among *TP53*-mutant HCCs (7/114, 6.1% vs. 4/244, 1.6%, *P* = 0.042, Fisher’s exact test).

We further compared the mutational signatures between HCCs with missense or deleterious *TP53* mutations. Interestingly, while 18.6% (13/70) of samples with missense *TP53* mutations displayed substantial contribution from signature 12, only 4.5% (2/44) of samples harboring deleterious *TP53* mutations did (*P* = 0.044, Fisher’s exact test), with signature 12 being the dominant signature in 15.7% (11/70) and 2.3% (1/44) of samples with missense or deleterious *TP53* mutations, respectively (*P* = 0.027, Fisher’s exact test, **Figure 4**). No difference in other signatures was observed. The aflatoxin-associated signature 24 was enriched among R249S-mutant HCCs compared other *TP53*-mutant HCCs (4/11, 36% vs. 3/103, 3%, *P* = 0.001 for substantial contribution and 3/11, 27% vs. 2/103, 2%, *P* = 0.006 for dominant signature, Fisher’s exact tests).

Taken together, our results suggest that the different types of *TP53* mutations were associated with distinct mutational processes. Specifically, signature 12 was rarely found in HCCs with deleterious *TP53* mutations.

### Distinct Types of *TP53* Mutations Are Associated with Different Prognoses

**FIGURE 5:**
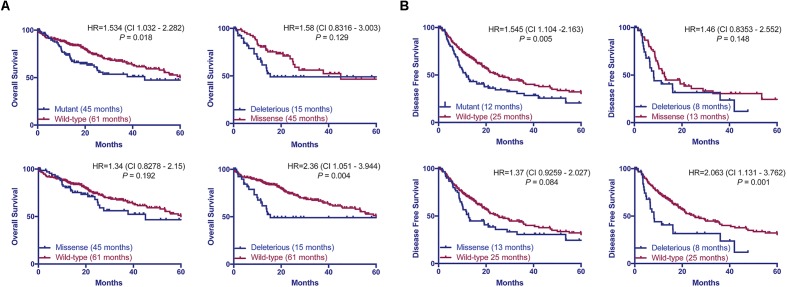
*TP53* mutation status is associated with worse overall and disease-free survival. Overall **(A)** and disease-free survival **(B)** of HCC patients with and without *TP53* somatic mutations using the Kaplan–Meier method. Median survival for each group is indicated in parentheses. Statistical comparisons were performed using log-rank tests. *P* < 0.05 was considered statistically significant.

Previous studies found that associations between the types of *TP53* mutations and prognoses in breast, and head and neck cancers (Olivier et al., 2006; Ozcelik et al., 2007; Vegran et al., 2013; Lapke et al., 2016). Here we hypothesized that patients with HCCs harboring *TP53* missense or deleterious mutations may display different prognoses. Considering the patients with available data on OS (*n* = 372) or DFS (*n* = 321), we found that patients with *TP53*-mutant HCCs displayed a more aggressive behavior including shorter OS and DFS than *TP53*-wild-type patients (*P* = 0.018 and *P* = 0.005, respectively, log-rank tests, **Figure 5**). Patients with missense or deleterious *TP53* mutations did not differ in OS or DFS (*P* = 0.129 and *P* = 0.148, respectively, log-rank tests, **Figure 5**). Importantly, while patients with deleterious *TP53* mutations had worse OS and DFS than *TP53*-wild-type patients (*P* = 0.004 and *P* = 0.001, respectively, log-rank tests, **Figure 5**), there was no difference in OS or DFS between patients with missense *TP53* mutations and those wild-type for *TP53* (*P* = 0.192 and *P* = 0.084, respectively, log-rank tests, **Figure 5**).

As an exploratory analysis, we asked whether OSCs or mutational signatures of *TP53*-mutant HCCs were prognostic. Compared to OSC1 (28 months), OSC2 (26 months) and OSC3 (median not reached), OSC4 was associated with the shortest median OS of 14 months, although the difference was not statistically significant (*P* = 0.366, log-rank test; Supplementary Figure S4E). Univariate Cox regression analyses revealed that the aflatoxin-associated signature 24 (HR 3.275, CI 1.279–8.384, *P* = 0.013), HBV infection status and the presence of necrotic areas were associated with poor prognosis (Supplementary Table S5). However, in a multivariate analysis, mutational signature 24 was not an independent prognostic indicator (*P* = 0.242; Supplementary Table S5).

Taken together, our results showed only patients with deleterious *TP53* mutations but not missense *TP53* mutations were associated with significantly worse OS and DFS in this cohort.

## Discussion

In this study, we performed a detailed analysis of *TP53* somatic mutational spectrum in HCCs, with nearly all missense mutations (98%) and most deleterious mutations (73%) affecting the DNA-binding domain. Notably, we found that the residues mutated in HCCs differed from those in other cancer types. Hotspot mutations R249S and V157F were common in HCCs but extremely rare in other cancers, while mutations affecting R175 and R273, two of the most frequently mutated residues in other cancers, were nearly absent in HCCs. This latter observation also applies to other HCC datasets (Ahn et al., 2014; Schulze et al., 2015), suggesting that *TP53* mutational spectrum in HCC is distinct from that in other cancers.

To determine the genotype–phenotype correlation between *TP53* mutation status and clinicopathologic parameters, we performed a detailed assessment of histologic features using H&E slides. We confirmed the established associations with the male gender, HBV/HCV infection and high Edmondson grade. Additionally, *TP53* mutations were associated with the presence of necrotic areas, and accordingly, with the absence of cholestasis, a feature more frequently observed in well-differentiated HCCs. Finally, we observed that the presence of TILs was associated with less frequent *TP53* mutations, in line with the favorable prognosis associated with tumors with high TILs in other tumor types (Mahmoud et al., 2011).

Analysis of the mutational signatures revealed that signatures 16 of unknown etiology and the age-associated signature 5 (Alexandrov et al., 2015) were the most prevalent in HCCs. We also found that signature 12 of unknown etiology, characterized by frequent T>C substitutions, was prevalent in *TP53*-wild-type and HCCs with missense *TP53* mutations but were largely absent in those with deleterious *TP53* mutation. A previous study reported that the W3 signature, which was highly similar to signature 12 (Fujimoto et al., 2012), was associated with the age of patients. Here we found no difference in the age of patients when we considered tumors with strictly missense or deleterious *TP53* mutations (i.e., excluding one patient with both types). The basis of signature 12 is thus unclear and further studies are required to elucidate its biological significance.

Adopting the algorithm of “oncosign” (Ciriello et al., 2013), we identified four robust subclasses of *TP53*-mutant HCCs with distinct oncogenic signatures. Of these classes, one subclass was likely driven by co-occurring *CTNNB1* mutations, while two subclasses were likely driven by amplicon drivers on 1q and 8q. 1q21 amplification has been linked to hepatocarcinogenesis, with *ALC1* (*CHD1L*) overexpression in HCC cells shown to promote G1/S phase transition and to inhibit apoptosis (Ma et al., 2008). The authors further suggested that the oncogenic function of ALC1 might be associated with its role in promoting cell proliferation by down-regulating p53 expression (Ma et al., 2008). The 1q21 amplicon also contains *HORMAD1*, a gene that has been shown to drive chromosomal instability in breast cancer (Watkins et al., 2015). As for 8q24, in addition to the well-known oncogenic role of *MYC*, previous studies have also shown that *MYC* amplification is an indicator of malignant potential and poor prognosis in HCC (Lin et al., 2010), and that the co-occurrence of *MYC* amplification and p53 alteration may contribute to HCC progression (Kawate et al., 1999). The remaining subclass did not have highly recurrent genetic alterations. Interestingly, this subclass was numerically, though not statistically, associated with the most favorable OS among the four classes. One may speculate that *TP53*-mutant HCCs lacking additional drivers may constitute a less aggressive subclass. Of note, the features that characterized the four OSCs were largely mutually exclusive, suggesting that distinct oncogenic processes are operative in non-overlapping subsets of *TP53*-mutant HCCs.

*TP53* mutation status predicts worse OS and DFS in HCC patients (Yano et al., 2007; Woo et al., 2011; Cleary et al., 2013). However, we found that patients with deleterious mutations, but not those with missense mutations, were associated with worse OS and DFS compared to patients wild-type for *TP53*. This is in line with other tumor types, in which different types of *TP53* mutations have been associated with different prognoses (Olivier et al., 2006; Ozcelik et al., 2007; Vegran et al., 2013; Lapke et al., 2016). In fact, the risk of death or relapse for patients harboring deleterious mutation is 2.3 times (HR = 2.36 and 2.063, respectively) higher than *TP53*-wild-type patients. The prognosis for patients with missense mutations appears to sit between those with wild-type *TP53* or deleterious *TP53* mutations, albeit not statistically different from either group. It is conceivable that the prognostic significance of the type of *TP53* mutations may be confirmed in a larger cohort with extensive follow-up.

It has been suggested that *TP53* missense mutations have varying capacity to transactivate p53 target genes and to alter the responsiveness to chemotherapeutic agents in breast cancer (Jordan et al., 2010). A differential expression analysis using the HCC TCGA dataset comparing HCCs with *TP53* missense mutations and those with *TP53* deleterious mutations identified *TP53* itself as up-regulated but did not identify significantly altered genes (data not shown). Furthermore, HCCs harboring the missense mutations functionally shown to lack the ability to transactivate genes with p53 response elements (Jordan et al., 2010) did not differ from HCCs with other missense mutations on the transcriptomic level (data not shown). It is thus unclear precisely how the various *TP53* mutations may differentially alter the transcriptomic landscape of HCCs. Further functional studies may be required to elucidate how the types of *TP53* mutations may affect its biological functions.

In HCC molecular characterization studies to date, HCCs are typically classified as *TP53*-wild-type or *TP53*-mutant, where all *TP53* mutations were treated as equal (Fujimoto et al., 2012; The Cancer Genome Atlas Research Network, 2017). However, many studies have demonstrated that *TP53* can be affected by either (or both) gain-of-function or loss-of-function mutations, with missense mutations preferentially displaying gain-of-function or neomorphic properties (Muller and Vousden, 2014). Our study has demonstrated that HCCs with missense or deleterious *TP53* mutations display similar clinicopathologic features, mutational/CNA burden and oncogenic signatures, but are associated with distinct mutational signatures. Clinically, while patients with tumors harboring deleterious *TP53* mutations had worse prognosis compared to those wild-type for *TP53*, there was no statistically significant difference between those with missense mutations and those wild-type for *TP53*. Our study highlights the importance to consider the type of *TP53* mutations in studies of biomarkers and molecular characterization of HCCs.

Our study has limitations. Despite TCGA being the largest genomic study of HCC, it is by no means the only large-scale study. However, as one of our aims was to define clinicopathologic correlates, we chose TCGA as it is the only study with publicly available H&E slides for pathology review. Secondly, the power of the OS and DFS analyses was limited due to the cohort size. Further studies may reveal whether prognosis is related to the type of *TP53* mutations, as has been shown in other cancers. Thirdly, our analyses did not consider the non-coding genome due to the nature of the sequencing performed by the TCGA. Given the frequent mutations in non-coding regions such as *TERT* promoter, *MALAT1* and *NEAT1* (Fujimoto et al., 2012; Schulze et al., 2015), it is conceivable that additional oncogenic signatures within *TP53*-mutant HCCs may emerge.

## Conclusion

Our study highlights the genetic heterogeneity among *TP53*-mutant HCCs and that patients with HCCs harboring different types of *TP53* mutations may be associated with distinct prognoses. Future work will be required to elucidate whether the co-occurring genetic alterations act synergistically with *TP53* mutations to promote carcinogenesis in HCCs.

## Author Contributions

CKYN and SP conceived and supervised the study. SA, MSM, and LMT performed the histologic review. VK performed the bioinformatics analyses under supervision of CKYN. VK, CKYN, and SP performed the statistical analyses. VK performed the transcriptomic classification under supervision of YH. VK, ML, LQ, GR, LMT, CKYN, and SP analyzed the data and critically discussed the results. CKYN and SP wrote the manuscript. All authors reviewed and approved the final version of the manuscript.

## Conflict of Interest Statement

The authors declare that the research was conducted in the absence of any commercial or financial relationships that could be construed as a potential conflict of interest.

## Footnotes

1 http://www.cbioportal.org

2 https://xenabrowser.net/datapages/?cohort=TCGA%20Liver%20Cancer%20(LIHC)
