# Supplementary material for: Genomic Analysis Revealed New Oncogenic Signatures in TP53-Mutant Hepatocellular Carcinoma
Source: Front Genet. 2018 Feb 2;9:2. doi: 10.3389/fgene.2018.00002 (PMC5801302; doi:10.3389/fgene.2018.00002)
Supplement: Supplementary file 1 [file Data_Sheet_1.pdf]

Supplementary Materials

Genomic Analysis Revealed New Oncogenic Signatures in *TP53*-mutant Hepatocellular Carcinoma

Venkatesh Kancherla[1], Samir Abdullazade[1], Matthias S Matter[1], Manuela Lanzafame[1], Luca Quagliata[1], Guglielmo Roma[2], Yujin Hoshida[3], Luigi M Terracciano[1], Charlotte KY Ng[1,4*] and Salvatore Piscuoglio[1*]

[*]**Correspondence to:**

Dr. Salvatore Piscuoglio (Salvatore.Piscuoglio@usb.ch) and **Dr. Charlotte K Y Ng** (kiuyancharlotte.ng@usb.ch), Institute of Pathology, University Hospital Basel, Schoenbeinstrasse 40, 4031 Basel, Switzerland. Tel: +41613286874; Fax: +41612653194.

- **Supplementary Methods**
- **Supplementary Figures S1 – S4**
- **Supplementary Tables S1 – S5**

Supplementary Methods

Classification of *TP53* somatic mutations

TP53 mutations were stratified according to (i) the mutation type as single-nucleotide missense mutations (encompassing missense, and synonymous mutations affecting splice-region) or deleterious mutations (encompassing splice site, nonsense, in-frame, and frameshift mutations); (ii) whether the mutations were within or outside of the DNA-binding domain.

The effects of missense mutations and synonymous mutations affecting splice-regions were predicted using CHASM (Carter et al., 2009), FATHMM (Shihab et al., 2013), VEST (Carter et al., 2013), MutationTaster (Schwarz et al., 2010) and PolyPhen-2 (Adzhubei et al., 2010). Annotation of ClinVar (Landrum et al., 2016), 1000 Genomes (Genomes Project et al., 2015), ESP6500 (Tennessen et al., 2012), ExAC (Lek et al., 2016) and COSMIC (Forbes et al., 2011) allele frequencies were retrieved from cravat.us (Douville et al., 2013). The two synonymous mutations affecting splice-region were included as missense mutations in the current study as both were predicted to be disease causing by MutationTaster (Schwarz et al., 2010).

Oncogenic signatures

Oncogenic signature (“oncosign”) classification was performed using the algorithm described by Ciriello *et al.* (Ciriello et al., 2013). The approach to select genomic features as ‘selected functional elements’ (SFEs) input data was adopted from Ciriello *et al.* (Ciriello et al., 2013). Specifically, from the 76 significantly mutated genes defined by MutSigCV obtained from the cbioportal (Gao et al., 2013), we selected 29 that have previously been reported as cancer genes by any of Cancer Gene Census (Futreal et al., 2004), Kandoth *et al.* (Kandoth et al., 2013), Lawrence *et al.* (Lawrence et al., 2014) or Fujimoto *et al.* (Fujimoto et al., 2012). Somatic mutation data were coded as a binary gene-by-sample matrix, with 1 indicating the presence of

one (or more) non-synonymous somatic mutation in a given gene in a given sample. GISTIC peaks were also selected as SFEs, and included 27 amplification and 34 deletion peaks, and were coded as a binary peak-by-sample matrix, with 1 indicating the presence of at least one gene harboring high-level gain/ amplification (copy number state '2' for amplification peaks) or homozygous deletion (copy number state '-2' for deletion peaks). The oncosign algorithm was run with a maximum depth of 1.

Pathway analysis

For Ingenuity Pathway Analysis (IPA), genes of interest were mapped to pathways and networks available in the Ingenuity database and ranked by corrected *P* value (Benjamini–Hochberg multiple correction) as previously described (Piscuoglio et al., 2014;Martelotto et al., 2015). $P<0.001$ was considered significant.

Statistical Analysis

For statistical analyses comparing copy number profiles, gene-level copy number states (i.e. amplification/high-level gains, gains, losses and homozygous deletions) were compared using Fisher's exact tests corrected for multiple comparisons using the Benjamini-Hochberg method (Piscuoglio et al., 2014). To define genes up-regulated when gained or amplified and genes down-regulated when lost, we applied Mann-Whitney U tests using categorical copy number states (i.e. gain vs. no gain, loss vs. no loss) as the grouping variable and the expression of genes as the dependent variable corrected for multiple comparisons using the Benjamini-Hochberg method.

Supplementary References

Adzhubei, I.A., Schmidt, S., Peshkin, L., Ramensky, V.E., Gerasimova, A., Bork, P., et al. (2010). A method and server for predicting damaging missense mutations. *Nat Methods* 7**,** 248-249.

Carter, H., Chen, S., Isik, L., Tyekucheva, S., Velculescu, V.E., Kinzler, K.W., et al. (2009). Cancer-specific high-throughput annotation of somatic mutations: computational prediction of driver missense mutations. *Cancer Res* 69**,** 6660-6667.

Carter, H., Douville, C., Stenson, P.D., Cooper, D.N., and Karchin, R. (2013). Identifying Mendelian disease genes with the variant effect scoring tool. *BMC Genomics* 14 Suppl 3**,** S3.

Ciriello, G., Miller, M.L., Aksoy, B.A., Senbabaoglu, Y., Schultz, N., and Sander, C. (2013). Emerging landscape of oncogenic signatures across human cancers. *Nat Genet* 45**,** 1127-1133.

Douville, C., Carter, H., Kim, R., Niknafs, N., Diekhans, M., Stenson, P.D., et al. (2013). CRAVAT: cancer-related analysis of variants toolkit. *Bioinformatics* 29**,** 647-648.

Forbes, S.A., Bindal, N., Bamford, S., Cole, C., Kok, C.Y., Beare, D., et al. (2011). COSMIC: mining complete cancer genomes in the Catalogue of Somatic Mutations in Cancer. *Nucleic Acids Res* 39**,** D945-950.

Fujimoto, A., Totoki, Y., Abe, T., Boroevich, K.A., Hosoda, F., Nguyen, H.H., et al. (2012). Whole-genome sequencing of liver cancers identifies etiological influences on mutation patterns and recurrent mutations in chromatin regulators. *Nat Genet* 44**,** 760-764.

Futreal, P.A., Coin, L., Marshall, M., Down, T., Hubbard, T., Wooster, R., et al. (2004). A census of human cancer genes. *Nat Rev Cancer* 4**,** 177-183.

Gao, J., Aksoy, B.A., Dogrusoz, U., Dresdner, G., Gross, B., Sumer, S.O., et al. (2013). Integrative analysis of complex cancer genomics and clinical profiles using the cBioPortal. *Sci Signal* 6**,** pl1.

Genomes Project, C., Auton, A., Brooks, L.D., Durbin, R.M., Garrison, E.P., Kang, H.M., et al. (2015). A global reference for human genetic variation. *Nature* 526**,** 68-74.

Kandoth, C., Mclellan, M.D., Vandin, F., Ye, K., Niu, B., Lu, C., et al. (2013). Mutational landscape and significance across 12 major cancer types. *Nature* 502**,** 333-339.

Landrum, M.J., Lee, J.M., Benson, M., Brown, G., Chao, C., Chitipiralla, S., et al. (2016). ClinVar: public archive of interpretations of clinically relevant variants. *Nucleic Acids Res* 44**,** D862-868.

Lawrence, M.S., Stojanov, P., Mermel, C.H., Robinson, J.T., Garraway, L.A., Golub, T.R., et al. (2014). Discovery and saturation analysis of cancer genes across 21 tumour types. *Nature* 505**,** 495-501.

Lek, M., Karczewski, K.J., Minikel, E.V., Samocha, K.E., Banks, E., Fennell, T., et al. (2016). Analysis of protein-coding genetic variation in 60,706 humans. *Nature* 536**,** 285-291.

Martelotto, L.G., De Filippo, M.R., Ng, C.K., Natrajan, R., Fuhrmann, L., Cyrta, J., et al. (2015). Genomic landscape of adenoid cystic carcinoma of the breast. *J Pathol* 237**,** 179-189.

Piscuoglio, S., Ng, C.K., Martelotto, L.G., Eberle, C.A., Cowell, C.F., Natrajan, R., et al. (2014). Integrative genomic and transcriptomic characterization of papillary carcinomas of the breast. *Mol Oncol* 8**,** 1588-1602.

Schwarz, J.M., Rodelsperger, C., Schuelke, M., and Seelow, D. (2010). MutationTaster evaluates disease-causing potential of sequence alterations. *Nat Methods* 7**,** 575-576.

Shihab, H.A., Gough, J., Cooper, D.N., Stenson, P.D., Barker, G.L., Edwards, K.J., et al. (2013). Predicting the functional, molecular, and phenotypic consequences of amino acid substitutions using hidden Markov models. *Hum Mutat* 34**,** 57-65.

Tennessen, J.A., Bigham, A.W., O'connor, T.D., Fu, W., Kenny, E.E., Gravel, S., et al. (2012). Evolution and functional impact of rare coding variation from deep sequencing of human exomes. *Science* 337**,** 64-69.

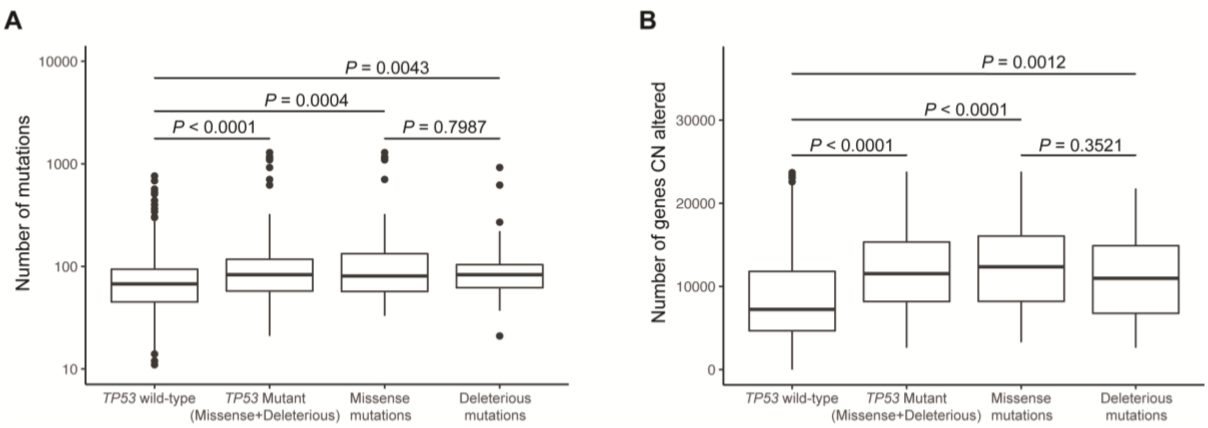

Supplementary Figure S1: Number of mutations (**A**) and copy number altered genes (**B**) identified in hepatocellular carcinomas stratified according to the *TP53* mutation status. Statistical comparisons were performed using Mann-Whitney U tests. *P*<0.05 was considered statistically significant.

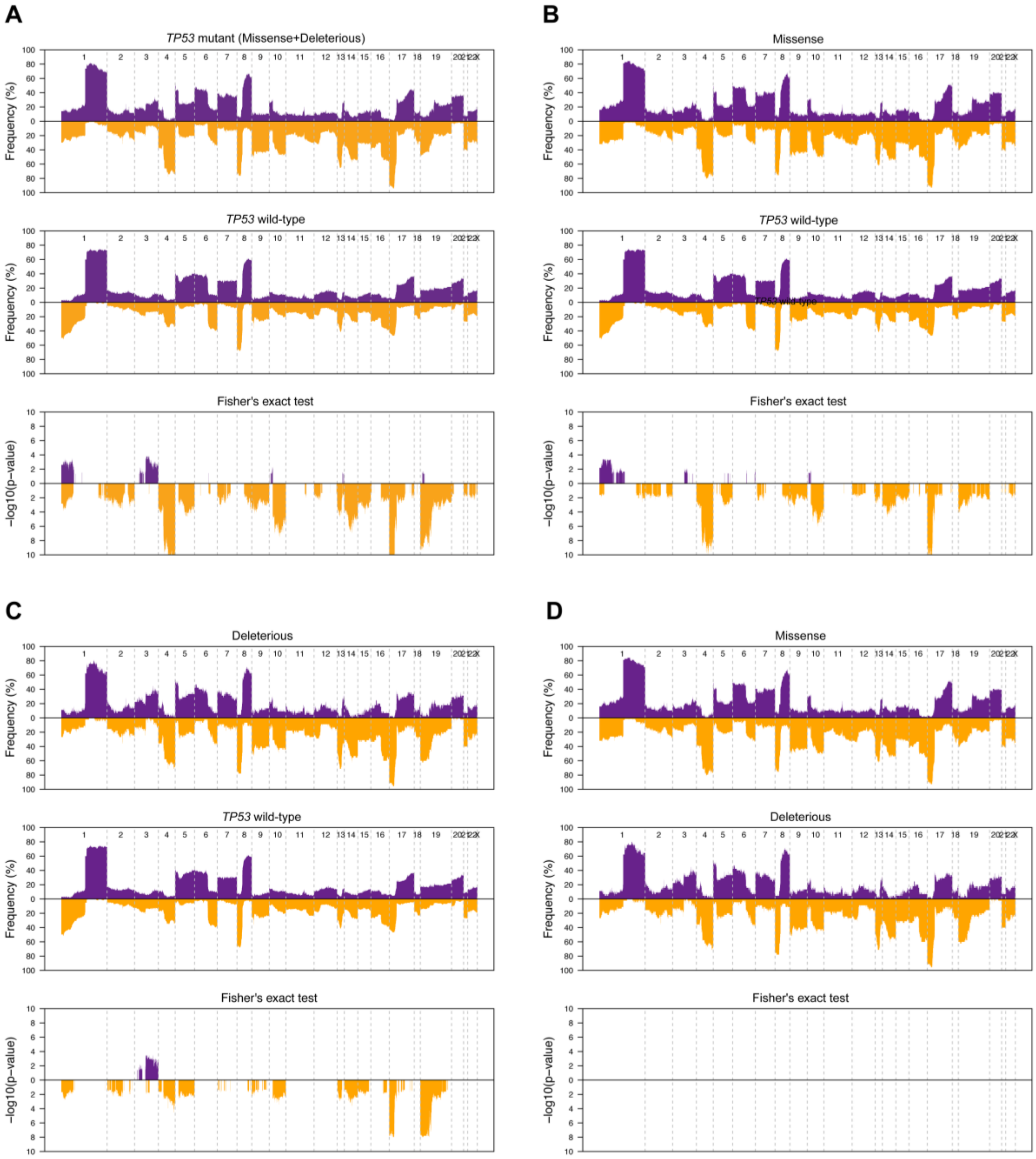

Supplementary Figure S2: Comparative genomic profiling of *TP53*-mutant and *TP53*-wild-type HCCs (**A-D**). Frequency plots and multi-Fisher's exact test comparisons of chromosomal gains and losses in *TP53*-mutant (top) and *TP53*-wild-type (middle) HCCs. The frequency of gains (purple bars) or losses (yellow bars) for each gene is plotted on the *y*-axis, according to their genomic position on the *x*-axis. Inverse Log_{10}values of the Fisher's exact test *P* values are plotted according to genomic location (*x*-axis) (bottom).

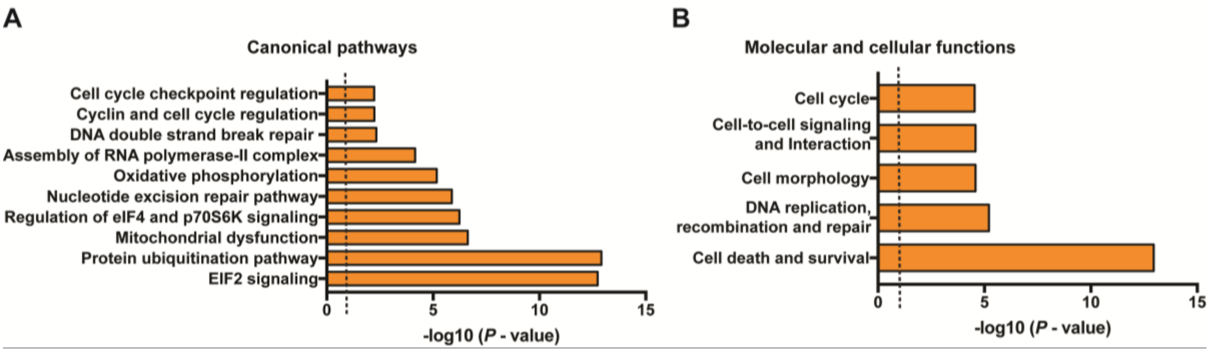

Supplementary Figure S3: Signaling pathways (left) and molecular and cellular functions (right) enriched among genes overexpressed when gained or downregulated when lost in the regions that showed differential CNA frequencies between cases with or without *TP53* mutations using Ingenuity Pathway Analysis (IPA). Log values of the Benjamini-Hochberg corrected *P* value are shown. Dashed lines indicate the significance cut-off ($P = 0.001$).

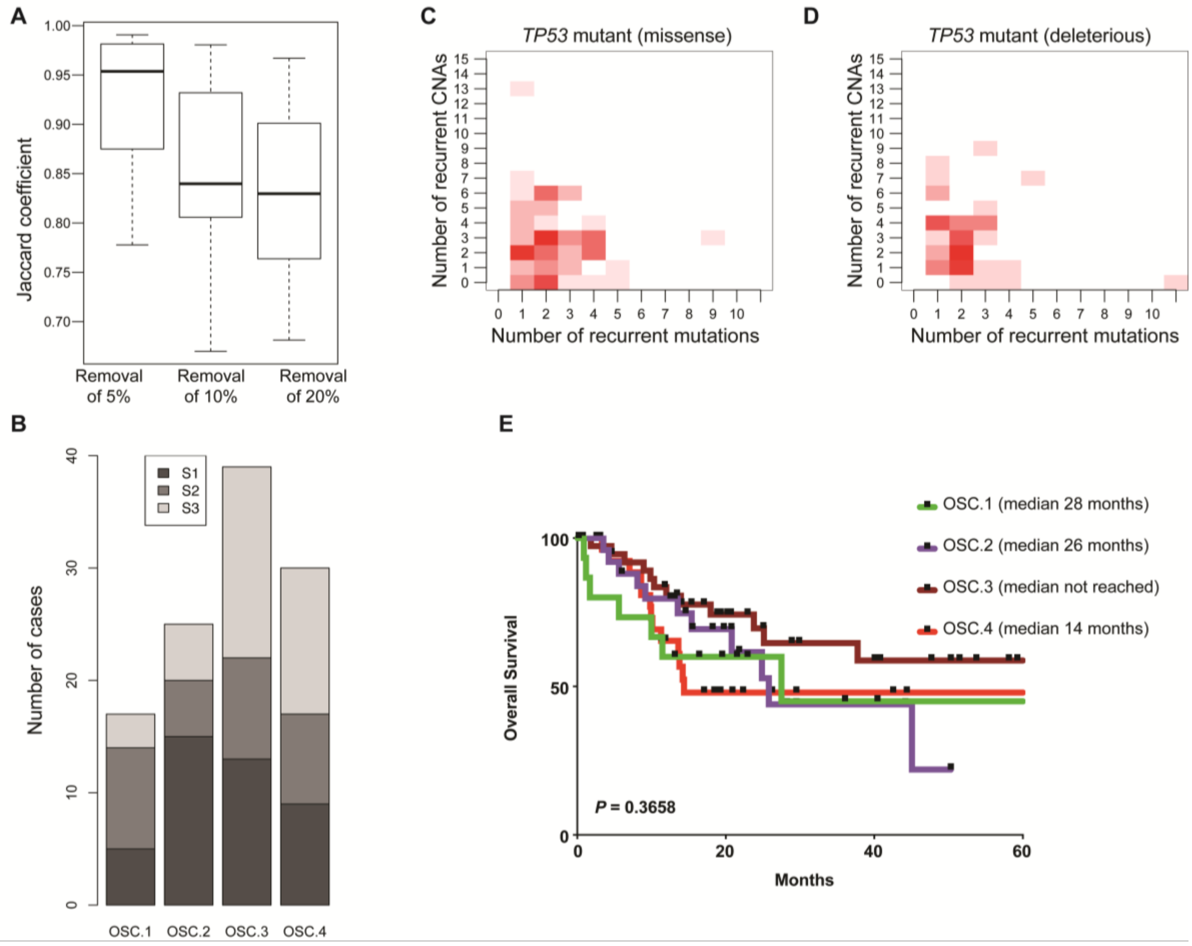

Supplementary Figure S4: Oncogenic signature subclasses were tested for robustness (**A**) upon removal of 5%, 10%, and 20% of the samples**.** Barplot shows the distribution of cases classified as S1, S2 or S3 based on transcriptomic classification among the four oncogenic signature classes (**B**). The distribution of mutational vs copy number 'selected functional elements' (SFEs) in *TP53*-mutant cases harboring missense (**C**) or deleterious (**D**) somatic *TP53* mutations. The shade of red is proportional to the number of samples for a given (*x*,*y*) position. Survival analysis of HCCs sub-classified based on the oncogenic signatures (**E**). Median survival for each group is indicated in parentheses. Statistical comparisons were performed using log-rank tests. $P < 0.05$ was considered statistically significant.

Supplementary Table S1: *In silico* prediction of mutation effect of missense and synonymous mutations affecting splice-regions.

Chrom	Position	Ref. base(s)	Alt. base(s)	Sequence ontology	Protein sequence change	CHASM viral liver cancer driver p-value (missense)	CHASM non-viral liver cancer driver p-value (missense)	VEST pathogenicity p-value (non-silent)	FATHMM	Polyphen	Mutation Taster	ClinVar Clinical Significance	1000 Genomes allele frequency	ESP6500 allele frequency (average)	ExAC total allele frequency	COSMIC ID	Occurrences in COSMIC	Number of samples in current study having the exact nucleotide change
chr17	7574006	A	C	missense	F341V	0.0786	0.2032	0.08944297	CANCER	benign	disease causing	.	0	0.00	0.00	.	.	1
chr17	7574033	T	A	missense	I332F	0.1313	0.1536	0.01250980	CANCER	probably damaging	disease causing	.	0	0.00	0.00	.	.	1
chr17	7577082	C	T	missense	E286K	0.0012	0.0020	0.00779240	CANCER	probably damaging	disease causing	Pathogenic	0	0.00	0.00	COSM10726	89	1
chr17	7577095	G	C	missense	D281E	0.0002	0.0020	0.00662004	CANCER	probably damaging	disease causing	.	0	0.00	0.00	COSM43837	21	1
chr17	7577099	C	T	missense	R280K	0.0008	0.0022	0.00644822	CANCER	probably damaging	disease causing	.	0	0.00	0.00	COSM10728	64	1
chr17	7577111	G	C	missense	A276G	0.0036	0.0082	0.01375201	CANCER	probably damaging	disease causing	.	0	0.00	0.00	COSM45695	5	1
chr17	7577114	C	T	missense	C275Y	0.0000	0.0002	0.00522482	CANCER	probably damaging	disease causing	.	0	0.00	0.00	COSM10893	59	1
chr17	7577115	A	G	missense	C275R	0.0000	0.0000	0.00536404	CANCER	probably damaging	disease causing	.	0	0.00	0.00	COSM43902	10	1
chr17	7577121	G	T	missense	R273S	0.0002	0.0012	0.00539233	CANCER	probably damaging	disease causing	.	0	0.00	0.00000889	COSM43909	15	1
chr17	7577126	T	A	missense	E271V	0.0050	0.0058	0.00615009	CANCER	probably damaging	disease causing	.	0	0.00	0.00	COSM44469	8	1
chr17	7577141	C	A	missense	G266V	0.0018	0.0012	0.00565372	CANCER	probably damaging	disease causing	.	0	0.00	0.00	COSM10958	49	1
chr17	7577142	C	T	missense	G266R	0.0020	0.0018	0.00683229	CANCER	probably damaging	disease causing	.	0	0.00	0.00	COSM10794	40	1
chr17	7577509	C	T	missense	E258K	0.0008	0.0032	0.00686832	CANCER	probably damaging	disease causing	Pathogenic	0	0.00	0.00000825	COSM10988	46	1
chr17	7577511	A	G	missense	L257P	0.0002	0.0020	0.00530791	CANCER	probably damaging	disease causing	.	0	0.00	0.00	COSM43842	12	1
chr17	7577524	T	C	missense	T253A	0.0018	0.0072	0.02753483	CANCER	probably damaging	disease causing	.	0	0.00	0.00	COSM45322	3	1
chr17	7577534	C	A	missense	R249S	0.0002	0.0020	0.00599047	CANCER	probably damaging	disease causing	Pathogenic	0	0.00	0.00	COSM10817	337	11
chr17	7577538	C	T	missense	R248Q	0.0020	0.0010	0.01018981	CANCER	probably damaging	disease causing	Likely pathogenic;Pathogenic	0	0.00005814	0.00005768	COSM10662	702	3
chr17	7577539	G	A	missense	R248W	0.0008	0.0020	0.00672533	CANCER	probably damaging	disease causing	Likely pathogenic;Pathogenic	0	0.00	0.00000824	COSM10656	592	1
chr17	7577545	T	C	missense	M246V	0.0018	0.0046	0.01627276	CANCER	probably damaging	disease causing	Likely pathogenic;Pathogenic	0	0.00	0.00	COSM43555	38	1
chr17	7577547	C	T	missense	G245D	0.0004	0.0036	0.00559456	CANCER	probably damaging	disease causing	Pathogenic	0	0.00	0.00	COSM43606	130	1
chr17	7577557	A	T	missense	C242S	0.0004	0.0020	0.00679644	CANCER	probably damaging	disease causing	.	0	0.00	0.00	COSM44935	15	1
chr17	7577565	T	C	missense	N239S	0.0018	0.0058	0.20211531	CANCER	probably damaging	disease causing	.	0	0.00	0.00	COSM44094	28	1
chr17	7577569	A	G	missense	C238R	0.0008	0.0012	0.00547809	CANCER	probably damaging	disease causing	.	0	0.00	0.00	COSM44321	18	1
chr17	7577570	C	A	missense	M237I	0.0020	0.0082	0.01231396	CANCER	probably damaging	disease causing	.	0	0.00	0.00	COSM11063	32	1
chr17	7577602	A	G	missense	S227P	0.0034	0.0082	0.01895411	CANCER	benign	disease causing	.	0	0.00	0.00	COSM1386652	3	1
chr17	7578190	T	C	missense	Y220C	0.0072	0.0134	0.00697755	CANCER	probably damaging	disease causing	Pathogenic	0	0.00	0.00002503	COSM10758	289	2
chr17	7578205	C	A	missense	S215I	0.0086	0.0612	0.00705133	CANCER	probably damaging	disease causing	.	0	0.00	0.00	COSM11450	23	1
chr17	7578205	C	T	missense	S215N	0.0163	0.0398	0.09182634	CANCER	probably damaging	disease causing	.	0	0.00	0.00	COSM44093	10	1
chr17	7578211	C	A	missense	R213L	0.0060	0.0354	0.00791633	CANCER	probably damaging	disease causing	.	0	0.00	0.00	COSM43650	47	1
chr17	7578211	C	T	missense	R213Q	0.0060	0.0014	0.01046134	CANCER	probably damaging	disease causing	Pathogenic	0	0.00	0.00000826	COSM10735	36	1
chr17	7578235	T	C	missense	Y205C	0.0044	0.0026	0.00621513	CANCER	probably damaging	disease causing	.	0	0.00	0.00	COSM43947	79	2
chr17	7578235	T	G	missense	Y205S	0.0062	0.0376	0.00553602	CANCER	probably damaging	disease causing	.	0	0.00	0.00	COSM44169	14	1
chr17	7578260	C	A	missense	V197L	0.0115	0.0618	0.01846215	CANCER	benign	disease causing	.	0	0.00	0.00	COSM46212	6	1
chr17	7578265	A	C	missense	I195S	0.0732	0.1454	0.00631397	CANCER	probably damaging	disease causing	.	0	0.00	0.00	COSM44539	9	1
chr17	7578268	A	C	missense	L194R	0.0135	0.0466	0.00565372	CANCER	probably damaging	disease causing	.	0	0.00	0.00	COSM44571	55	1
chr17	7578271	T	C	missense	H193R	0.0125	0.0102	0.00605381	CANCER	probably damaging	disease causing	.	0	0.00	0.00	COSM10742	100	4
chr17	7578402	G	C	missense	C176W	0.0012	0.0034	0.00982147	CANCER	probably damaging	disease causing	.	0	0.00	0.00	COSM11114	13	1
chr17	7578410	T	A	missense	R174W	0.0074	0.0094	0.03716059	CANCER	probably damaging	disease causing	.	0	0.00	0.00	COSM44782	12	1
chr17	7578449	C	A	missense	A161S	0.0024	0.0238	0.02902182	CANCER	probably damaging	disease causing	.	0	0.00	0.00	COSM43549	4	2
chr17	7578457	C	T	missense	R158H	0.0018	0.0018	0.00605381	CANCER	probably damaging	disease causing	Pathogenic	0	0.00	0.00000825	COSM10690	87	2
chr17	7578460	A	C	missense	V157G	0.0010	0.0046	0.00808464	CANCER	probably damaging	disease causing	.	0	0.00	0.00	COSM43903	10	1
chr17	7578461	C	A	missense	V157F	0.0014	0.0144	0.01231396	CANCER	probably damaging	disease causing	Pathogenic	0	0.00	0.00	COSM10670	183	3
chr17	7578463	C	G	missense	R156P	0.0060	0.0078	0.01905406	CANCER	probably damaging	polymorphism	.	0	0.00	0.00	COSM10760	27	1
chr17	7578478	G	T	missense	P151H	0.0010	0.0154	0.02105657	CANCER	probably damaging	disease causing	.	0	0.00	0.00	COSM11476	31	1
chr17	7578503	C	T	missense	V143M	0.0072	0.0396	0.02478553	CANCER	probably damaging	disease causing	.	0	0.00	0.00	COSM43878	26	1
chr17	7578513	C	A	missense	K139N	0.0072	0.0208	0.10473036	CANCER	probably damaging	disease causing	.	0	0.00	0.00	COSM44220	6	1
chr17	7578554	A	C	missense	Y126D	0.0042	0.0068	0.00550698	CANCER	probably damaging	disease causing	.	0	0.00	0.00	COSM43900	11	1
chr17	7578554	A	T	missense	Y126N	0.0034	0.0044	0.00912426	CANCER	probably damaging	disease causing	.	0	0.00	0.00	COSM44380	10	1
chr17	7579349	A	C	missense	F113C	0.0018	0.0162	0.00739314	CANCER	probably damaging	disease causing	.	0	0.00	0.00	COSM10717	9	1
chr17	7579369	G	C	missense	S106R	0.0404	0.1558	0.02544598	CANCER	benign	polymorphism	.	0	0.00	0.00	COSM45944	7	1
chr17	7579312	C	A	splice_region	T125T	N/A	N/A	N/A	N/A	N/A	disease causing	.	0	0.00	0.00	COSM45940	24	1
chr17	7578177	C	T	splice_region	E224E	N/A	N/A	N/A	N/A	N/A	disease causing	.	0	0.00	0.00	COSM44754	9	1

Supplementary Table S2: Detailed list of the TCGA studies included for the comparison of *TP53* mutational spectrum.

Study name	Number of cases altered	% of cases altered	Total number of the cases
Adrenocortical Carcinoma (TCGA, Provisional)	18	20.0	90
Acute Myeloid Leukemia (TCGA, Provisional)	16	8.1	197
Bladder Urothelial Carcinoma (TCGA, Provisional)	64	49.2	130
Breast Invasive Carcinoma (TCGA, Provisional)	301	30.7	982
Kidney Renal Clear Cell Carcinoma (TCGA, Provisional)	15	3.3	451
Cervical Squamous Cell Carcinoma and Endocervical Adenocarcinoma (TCGA, Provisional)	9	4.6	194
Cholangiocarcinoma (TCGA, Provisional)	5	14.3	35
Kidney Chromophobe (TCGA, Provisional)	22	33.3	66
Colorectal Adenocarcinoma (TCGA, Provisional)	120	51.5	233
Lymphoid Neoplasm Diffuse Large B-cell Lymphoma (TCGA, Provisional)	5	10.4	48
Esophageal Carcinoma (TCGA, Provisional)	153	82.7	185
Glioblastoma Multiforme (TCGA, Provisional)	84	29.0	290
Brain Lower Grade Glioma (TCGA, Provisional)	146	51.0	286
Head and Neck Squamous Cell Carcinoma (TCGA, Provisional)	366	71.5	512
Lung Adenocarcinoma (TCGA, Provisional)	106	46.1	230
Lung Squamous Cell Carcinoma (TCGA, Provisional)	128	72.3	177
Skin Cutaneous Melanoma (TCGA, Provisional)	56	15.2	368
Mesothelioma (TCGA, Provisional)	14	16.1	87
Ovarian Serous Cystadenocarcinoma (TCGA, Provisional)	275	87.0	316
Pancreatic Adenocarcinoma (TCGA, Provisional)	104	69.3	150
Pheochromocytoma and Paraganglioma (TCGA, Provisional)	1	0.5	184
Kidney Renal Papillary Cell Carcinoma (TCGA, Provisional)	7	2.5	282
Prostate Adenocarcinoma (TCGA, Provisional)	61	12.2	499
Sarcoma (TCGA, Provisional)	85	34.4	247
Stomach Adenocarcinoma (TCGA, Provisional)	190	48.1	395
Testicular Germ Cell Cancer (TCGA, Provisional)	2	1.3	155
Thymoma (TCGA, Provisional)	4	3.3	123
Thyroid Carcinoma (TCGA, Provisional)	3	0.7	405
Uterine Corpus Endometrial Carcinoma (TCGA, Provisional)	69	27.8	248
Uterine Carcinosarcoma (TCGA, Provisional)	52	91.2	57
Uveal Melanoma (TCGA, Provisional)	0	0.0	80

Supplementary Table S3: Clinicopathologic features of the 373 HCCs from The Cancer Genome Atlas cohort.

Parameters		N of samples	%
Age at diagnosis (n=372)	median 61 (range 16-90)		
Gender (n=372)	Female	121	32.5
	Male	251	67.5
Race (n=362)	American indian or Alaska native	2	0.6
	Asian	159	43.9
	Black or African American	17	4.7
	Caucasian	184	50.8
Risk factor (n=353)*	No History of Primary Risk Factors	91	25.8
	Alcohol consumption	117	33.1
	Hepatitis B virus	106	30.0
	Hepatitis C virus	56	15.9
	Hemochromatosis	7	2.0
	Non-Alcoholic Fatty Liver Disease	20	5.7
	Other	21	5.9
Child pugh classification grade (n=243)	A	221	90.9
	B	21	8.6
	C	1	0.5
Edmondson Grade (n=373)	1	0	0.0
	2	124	33.2
	3	226	60.6
	4	20	5.4
Cholestasis (n=370)**	Absent	290	78.4
	Present	80	21.6
Mallory Bodies (n=373)	Absent	291	78.0
	Present	82	22.0
Vessel infiltration (n=370)**	Absent	244	65.9
	Present	126	34.1
Necrotic areas (n=371)***	Absent	279	75.2
	Present	92	24.8
Infiltrating lymphocytes (372)****	Absent	196	52.7
	Present	176	47.3
Molecular classification by Hoshida et al. (n=367)*****	S1	115	31.3
	S2	79	21.5
	S3	179	47.2

* Patients may have multiple risk factors
** 3 cases were not evaluable.
*** 2 cases were not evaluable.
**** 1 cases were not evaluable.
***** 6 cases were not possible to classified.

Supplementary Table S4: Analyses of *TP53* mutation status sub-divided according to the mutation type and clinicopathologic parameters in the 373 HCCs from The Cancer Genome Atlas cohort. Statistical comparisons were performed using Fisher's exact test or Chi-Squared test. $P < 0.05$ was considered statistically significant.

		Type of Mutation		*P* value
		Deleterious	Missense	
**Age**	**Median years**	64	58	**0.049**
**Gender**	**Female**	8	17	0.491
	**Male**	37	53	
**Child pugh classification grade**	**A**	26	39	1.000
	**B**	3	4	
**Race Category**	**American indian or Alaska native**	0	1	0.837
	**Asian**	23	35	
	**Black or African American**	4	8	
	**Caucasian**	17	25	
**History of Primary Risk Factors**	**At least one risk factor**	35	57	1.000
	**No risk factor**	7	12	
**Edmondson Grade**	**2**	7	8	0.661
	**3**	32	55	
	**4**	6	7	
**Cholestasis**	**Absent**	39	62	0.777
	**Present**	6	8	
**Mallory Bodies**	**Absent**	34	60	0.217
	**Present**	11	10	
**Vessel infiltration**	**Absent**	28	44	1.000
	**Present**	17	26	
**Necrotic areas**	**Absent**	29	46	1.000
	**Present**	16	24	
**Infiltrating lymphocytes**	**Absent**	26	46	0.433
	**Present**	19	24	
**Molecular classification by Hoshida et al. (n=367)**	**S1**	14	28	0.459
	**S2**	11	20	
	**S3**	18	21	

Supplementary Table S5: Univariate and multivariate analyses of OS and DFS of TP53-mutant HCCs with clinicopathologic and molecular features.

	Univariate (Overall survival)				Multivariate (Overall survival)				Univariate (Disease -free survival)			
		Confidence Interval				Confidence Interval				Confidence Interval		
	HR	95% low	95% high	*P*	HR	95% low	95% high	*P*	HR	95% low	95% high	*P*
**Type of mutation**	0.630	0.343	1.156	0.136					0.667	0.393	1.130	0.132
**Hotspots**	1.699	0.883	3.270	0.113					1.283	0.715	2.302	0.405
**Gender**	0.961	0.461	2.005	0.916					1.152	0.624	2.129	0.651
**Caucasian**	1.361	0.739	2.505	0.322					1.598	0.952	2.682	0.076
**Black or African American**	0.997	0.356	2.792	0.996					0.392	0.096	1.604	0.193
**Asian**	0.787	0.431	1.437	0.435					0.722	0.433	1.204	0.212
**Alcohol consumption**	0.968	0.516	1.816	0.920					1.163	0.688	1.964	0.573
**Hepatitis B virus**	0.211	0.088	0.506	**0.000**	0.233	0.096	0.565	**0.001**	0.309	0.165	0.581	**0.000**
**Hepatitis C virus**	1.298	0.633	2.661	0.477					1.347	0.724	2.506	0.347
**Non-Alcoholic Fatty Liver Disease**	0.907	0.219	3.757	0.893					1.464	0.583	3.677	0.417
**Cholestasis**	0.561	0.173	1.816	0.335					0.604	0.242	1.512	0.282
**Mallory bodies**	0.793	0.352	1.785	0.575					0.842	0.427	1.662	0.621
**Infiltrating lymphocytes**	0.974	0.527	1.798	0.932					0.928	0.549	1.567	0.780
**Vascular invasion**	0.976	0.529	1.800	0.937					1.278	0.770	2.123	0.343
**Necrotic areas**	2.578	1.412	4.707	**0.002**	2.176	1.133	4.177	**0.020**	1.064	0.623	1.819	0.819
**Edmonson grade**	1.421	0.787	2.568	0.244					1.307	0.828	2.063	0.250
**OSC1**	1.355	0.603	3.046	0.463					0.934	0.412	2.117	0.871
**OSC2**	1.055	0.531	2.093	0.879					1.371	0.776	2.424	0.277
**OSC3**	0.579	0.297	1.130	0.109					0.759	0.446	1.290	0.308
**OSC4**	1.463	0.761	2.811	0.254					1.165	0.657	2.066	0.602
**Mutational sig 3**	0.702	0.169	2.915	0.626					0.983	0.354	2.726	0.973
**Mutational sig 5**	0.341	0.105	1.102	0.072					0.840	0.434	1.629	0.606
**Mutational sig 12**	1.202	0.507	2.851	0.676					1.248	0.590	2.640	0.563
**Mutational sig 16**	0.955	0.523	1.744	0.881					1.008	0.605	1.679	0.976
**Mutational sig 22**	1.251	0.386	4.050	0.709					1.120	0.349	3.592	0.849
**Mutational sig 24**	3.275	1.279	8.384	**0.013**	1.805	0.672	4.852	0.242	2.896	0.887	9.457	0.078

HR: Hazard Ratio
